# Influence of Environmental Factors on Biofilm Formation of Staphylococci Isolated from Wastewater and Surface Water

**DOI:** 10.3390/pathogens11101069

**Published:** 2022-09-20

**Authors:** Vanessa Silva, José Eduardo Pereira, Luís Maltez, Patrícia Poeta, Gilberto Igrejas

**Affiliations:** 1 Microbiology and Antibiotic Resistance Team (MicroART), Department of Veterinary Sciences, University of Trás-os-Montes and Alto Douro (UTAD), 5000-801 Vila Real, Portugal; 2 Department of Genetics and Biotechnology, University of Trás-os-Montes and Alto Douro (UTAD), 5000-801 Vila Real, Portugal; 3 Functional Genomics and Proteomics Unit, University of Trás-os-Montes and Alto Douro (UTAD), 5000-801 Vila Real, Portugal; 4 LAQV-REQUIMTE, Department of Chemistry, NOVA School of Science and Technology, Universidade Nova de Lisboa, 2829-516 Caparica, Portugal; 5 CECAV—Veterinary and Animal Research Centre, University of Trás-os-Montes and Alto Douro (UTAD), 5000-801 Vila Real, Portugal; 6 Associate Laboratory for Animal and Veterinary Science (AL4AnimalS), University of Trás-os-Montes and Alto Douro (UTAD), 5000-801 Vila Real, Portugal

**Keywords:** biofilms, environment, *Staphylococcus*, *S. aureus*, coagulase-negative staphylococci

## Abstract

The presence of biofilms can negatively affect several different areas, such as the food industry, environment, and biomedical sectors. Conditions under which bacteria grow and develop, such as temperature, nutrients, and pH, among others, can largely influence biofilm production. *Staphylococcus* species survive in the natural environment due to their tolerance to a wide range of temperatures, dryness, dehydration, and low water activity. Therefore, we aimed to evaluate the influence of external environmental factors on the formation of biofilm of staphylococci isolated from hospital wastewater and surface waters. We investigated the biofilm formation of methicillin-resistant and -susceptible *S. aureus* (MRSA and MSSA) and coagulase-negative staphylococci (CoNS) under various temperatures, pH values, salt concentrations, glucose concentrations, and under anaerobic and aerobic conditions. CoNS had the ability to produce more biofilm biomass than MSSA and MRSA. All environmental factors studied influenced the biofilm formation of staphylococci isolates after 24 h of incubation. Higher biofilm formation was achieved at 4% of NaCl and 0.5% of glucose for MSSA and CoNS, and 1% of NaCl and 1.5% of glucose for MRSA isolates. Biofilm formation of isolates was greater at 25 °C and 37 °C than at 10 °C and 4 °C. pH values between 6 and 8 led to more robust biofilm formation than pH levels of 9 and 5. Although staphylococci are facultative anaerobes, biofilm formation was higher in the presence of oxygen. The results demonstrated that multiple environmental factors affect staphylococci biofilm formation. Different conditions affect differently the biofilm formation of MRSA, MSSA, and CoNS strains.

## 1. Introduction

*Staphylococcus aureus* is a commensal organism and is typically not harmful to the host. However, it can breach innate host defenses and cause a wide range of infections [1]. The enormous health burden associated with *S. aureus* is partially attributed to its ability to acquire antimicrobial resistance determinants making infections very difficult to treat [2]. In contrast, coagulase-negative staphylococci (CoNS) were considered apathogenic. Nevertheless, recently, they have been progressively responsible for life-threatening infections in hospitals [3]. Methicillin-resistant *Staphylococcus aureus* (MRSA) and methicillin-resistant CoNS (MRCoNS) are one of the main pathogens in nosocomial infections leading to elevated morbidity and mortality [4,5]. Staphylococci are also often responsible for chronic infections due to their ability to form biofilms. Biofilms are structured aggregations of bacterial cells which attached to a biotic or abiotic surface proliferating and accumulating in multilayer cell clusters [6]. Biofilms are surrounded by a self-produced matrix which protects bacterial cells against environmental stresses, antimicrobials, disinfectants, and host immune defenses [7]. Biofilm mechanisms of resistance are distinct from the well-characterized intrinsic mechanisms that occur at the cellular level, being operated additively to those, in a transient and reversible manner, resulting in up to 1000-fold higher resistance levels [7,8]. Biofilm formation, together with staphylococci tolerance to dehydration, drying, and low water activity, justifies the widespread distribution and persistence of these bacteria in the natural environment [9].

Staphylococci are spread across the natural environment and have been isolated from air, dust, wild animals, and water [10,11,12,13]. Furthermore, several studies have reported the presence of MRSA and MRCoNS in wastewater, sea, river, and surface waters [13,14,15]. In fact, it has been shown that biofilm formation in piping and aquatic systems improved bacterial survival in the water environment [16]. However, environmental factors such as pH, temperature, nutrient content, salinity, and dissolved oxygen play important roles in the development of staphylococcal biofilm, influencing their persistence in water [6,17]. Staphylococci in wastewater may reach the natural aquatic ecosystems since it has been shown that wastewater treatment does not completely eliminate bacteria [14]. Once present in surface waters, staphylococci disperse in the environment, spreading to humans and animals entering the food chain [18]. Therefore, it is important to know and understand the impact of environmental factors that may influence biofilm formation. In this study, we aimed to characterize the biofilm formation capacity of staphylococci isolated from hospital wastewaters and surface waters and to investigate the influence of pH, NaCl, temperature, glucose, and oxygen on biofilm formation.

## 2. Material and Methods

### 2.1. Study Design

Part of this work was a retrospective study that included 112 staphylococci strains, comprising *mec*A-MRSA, *mec*C-MRSA, and methicillin-susceptible *S. aureus* (MSSA), CoNS, and MRCoNS isolates. The isolates were recovered from hospital wastewaters and surface waters between 2019 and 2020: four *mec*C-MRSA, 29 MSSA, 28 *S. sciuri*, five *S. lentus*, five *S. xylosus*, four *S. epidermidis*, two *S. urealyticus*, two *S. vitulinus*, one *S. caprae*, one *S. succinus*, one *S. carnous* spp. *carnous*, one *S. equorum* and one *S. simulans* from surface waters, and 28 *mec*A-MRSA from hospital wastewaters [13,14]. All isolates have been previously characterized regarding their antimicrobial resistance and *S. aureus* genetic lineages by MLST, *spa*-and *agr*-typing [13,14]. *S. aureus* ATCC^®^ 25923 was used as a positive control since it has a high ability to form biofilm.

### 2.2. Biofilm Formation

The biofilm formation was performed by the microtiter assay as previously described with some modifications [19]. Briefly, each staphylococcal isolate was streaked on brain heart infusion (BHI, Liofilchem, Rosetodegli, Abruzzi, Italy) agar plates and incubated at 37 °C for 24 h. After the incubation period, two staphylococcal colonies were transferred to tubes containing 3 mL of Tryptic Soy Broth (TSB, Oxoid Ltd., Basingstoke, UK) and incubated at 37 °C for 16 ± 1 h with continuous shaking at 120 rpm (ES-80 Shaker-incubator, Grant Instruments, Cambridge, UK). Then, the bacterial suspension was adjusted to an optical density of 1 × 10^6^ colony forming units and 200 µL of bacterial suspension was added to each well of the 96-well flat bottom microplate. S. *aureus* ATCC^®^ 25923 was included in all plates as a positive control. TSB without bacterial inoculum was used as a negative control. The plates were incubated at 37 °C for 24 h without shaking under aerobic conditions. All experiments had seven technical replicates.

#### 2.2.1. Biofilm Biomass Quantification

Biofilm biomass was quantified using the Crystal Violet (CV) Staining method as previously described by Peeters et al. (2008), with some modifications [20]. After incubation, the medium was carefully removed from each well and the plates were washed twice with distilled water to remove non-attached bacterial cells. The plates were allowed to dry at room temperature for 2 h. To fix the biofilms, 100 µL of methanol (VWR International) was added to each well and incubated for 15 min. Methanol was then removed, the plates were airdried at room temperature for 10 min and 100 µL of CV at 1% (*v*/*v*) was added to each well for 10 min. Then, the CV was removed, and the plates were washed twice with distilled water to remove the excess dye. Then, 100 µL of acetic acid 33% (*v*/*v*) was added to solubilize the CV and the absorbance was measured at 570 nm using a microplate reader BioTek ELx808U (BioTek, Winooski, VT, USA). To standardize the results, biofilm formation of each isolate was normalized according to the results obtained from the positive control strain *S. aureus* ATCC^®^ 25923.

### 2.3. Influence of Environmental Factors on Biofilm Formation

A total of 33 strains, representative of the bacterial collection, were used to investigate the influence of pH, temperature, glucose, salinity, and oxygen on the biofilm formation: 11 MRSA, 11 MSSA, and 11 CoNS. All isolates were seeded onto BHI plates and incubated at 37 °C for 24 h. Then, to evaluate the influence of glucose and salinity, TSB medium supplemented with 0.5%, 1%, 1.5%, 2%, and 2.5% of glucose and with 1%, 2%, 4%, and 8% of NaCl was prepared. TSB medium pH was adjusted by adding sodium hydroxide (Merck, Darmstadt, Germany) and hydrochloric acid (Merck, Darmstadt, Germany), and the studied range of pH was between 5 and 9 in step 1. Biofilm formation was carried out as described in Section 2.2. Overnight cultures of staphylococci were adjusted to 1 × 10^6^ colony-forming units and 20 µL of bacterial suspension and 180 μL of adjusted sterile TSB were added to each well of the 96-well flat bottom microplate. The plates were incubated at 37 °C for 24 h without shaking. All experiments had five technical replicates.

Biofilm formation was carried out as described in Section 2.2 to evaluate the effect of temperature and oxygen. Several plate replicates were used and were incubated for 24 h without shaking under different conditions: temperature at 4, 10, 25, 37, and 42 °C, and under aerobic and anaerobic conditions. For anaerobic conditions, the microplates were incubated at 37 °C for 24 h under anoxic conditions (Oxoid AnaeroGen System; ThermoFisher Scientific, Waltham, MA, USA) in an anaerobic jar. All experiments had five technical replicates. After the incubation period, the biofilm biomass was quantified as described in Section 2.2.1.

Control strain *S. aureus* ATCC^®^ 25923 was used in all experiments and was tested under all different conditions. To standardize the results, biofilm formation of each isolate was normalized according to the results obtained from the positive control strain.

### 2.4. Statistical Analysis

Descriptive statistics of data are presented as the mean (M) and standard deviation (SD) when appropriate. Skewness and kurtosis coefficients were computed for univariate normality analysis purposes. To analyze if the environmental conditions influenced the biofilm formation, ANOVA followed by Tukey’s or Dunnett’s tests was performed. All statistical analysis was performed using SPSS (version 26, IBM SPSS Statistics, Chicago, IL, USA). Statistically significant effects were assumed for *p* < 0.05.

## 3. Results and Discussion

Staphylococci are frequently present in the natural environment, including surface waters. These bacteria are known for their capacity to form biofilms which are found in water and wastewater treatment systems [21]. Hospital wastewater treatment plants (WWTPs) receive inputs of antibiotics and other drugs providing an ideal setting for the development of antimicrobial resistance and selection of antimicrobial resistant bacteria (ARB) [22]. Although studies have shown that wastewater treatment processes may reduce antimicrobial resistance genes (ARGs) and bacteria, they are not totally efficient and ARB and ARGs are released to the receiving water bodies through WWTPs effluents [23,24]. Bacteria in river water and other surface water may also form biofilms which provide an optimum environment for genetic exchange and accumulation of mobile genetic elements [25]. Once in the environment, biofilm-forming bacteria may impose a public health and environmental concern. Drinking water sources may also be a reservoir of ARB due to the link with antibiotic production wastewater, polluted river water, and hospital sewage [26]. In addition, biofilms in water distribution systems may be constituted by pathogens which may pose a public health concern [27,28,29]. Since biofilms play an important role in water and wastewater treatment plants, understanding the influence of environmental factors on biofilm formation may be essential to prevent the dissemination of pathogens through the environment and for drinking water biosafety.

**Figure 1:**
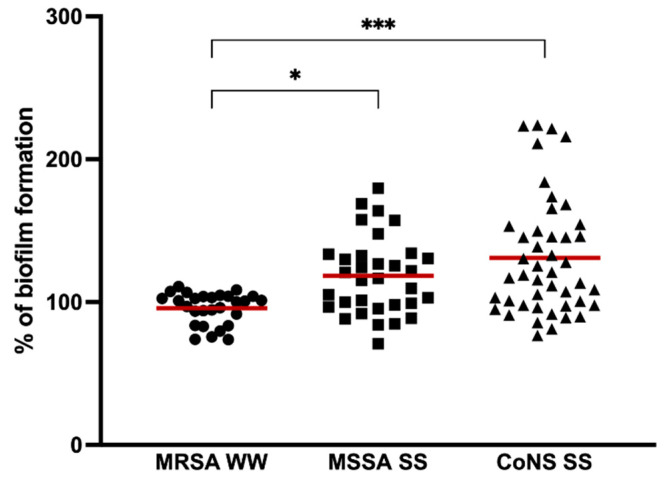
Biofilm formation capacity of MRSA isolated from hospital wastewater (MRSA WW), MSSA, and CoNS from surface water (MSSA, SS, and CoNS, respectively). The symbols represent the biomass mean of the biofilm formed by the individual isolates. The red lines represent the average of biofilm mass formed by all isolates. Statistical significance was determined using Tukey’s multiple comparison test (* *p* < 0.05; *** *p* < 0.001).

In our study, we investigated the biofilm-forming capacity of staphylococci strains isolated from hospital wastewater and surface waters. Biofilm formation was performed in all isolates by the microtiter assay. The percentage of biofilm biomass produced normalized against *S. aureus* ATCC 25923 is shown in Figure 1. The absorbance values are shown in Supplementary Table S1. All isolates were capable of biofilm formation. In a study by Ugwoke et al., staphylococci isolated from wastewater were also biofilm producers [30]. MRSA isolates from the hospital wastewater formed significantly weaker biofilms than MSSA isolated from surface waters (*p* < 0.05), with a percentage mean of 95.76 ± 11.03 and 118.29 ± 27.04, respectively. CoNS produced more biofilms biomass, with a percentage mean of 131.02 ± 41.07, when compared to *S. aureus* being significantly higher than MRSA isolates (*p* < 0.001). Although the biofilm formation of CoNS was higher than MSSA isolates, the differences were not significant. There are not many comparing the biofilm formation of *S. aureus* and CoNS isolated from water, but studies carried out with *S. aureus* and CoNS from cow’s milk revealed that a higher number of CoNS strains formed stronger biofilms when compared to *S. aureus* strains as happened in our study [31,32].

**Figure 2:**
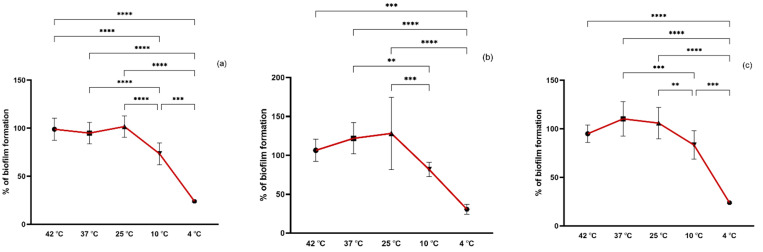
Biofilm formation capacity of MRSA (**a**), MSSA (**b**), and CoNS (**c**) under different temperatures. Symbols represent the biomass mean of the biofilm formed at each temperature tested. Statistical significance was determined using Tukey’s multiple comparison test (** *p* < 0.01; *** *p* < 0.001, **** *p* < 0.0001).

Environmental factors contributing to biofilm formation of staphylococci have been mostly studied in isolates from food since staphylococci is one of the most important pathogens involved in outbreaks of foodborne disease. However, the biofilm-forming capacity of staphylococci may vary with the origin, genetic lineages, and antimicrobial resistance, among others. Our isolates belong to important clinical and animal- and environmental-associated clonal lineages which may end up and spread in the environment reaching humans and animals through drinking water, agricultural fields, livestock, and even the food industry sector. In our study, we evaluated the biofilm formation of the isolates under different temperatures (4, 10, 25, 37, and 42 °C) to which isolates may be exposed in the environment. The optimal temperature for biofilm formation of *S. aureus* strains, both MRSA and MSSA, was 25 °C followed by 37 °C while for the CoNS isolates it was 37 °C (Figure 2). At 42 °C, a high biofilm formation capacity of all strains was also noted with no significant differences in biofilm formation between 25, 37, and 42 °C. However, at 10 °C, the biofilm formation of all isolates was significantly lower than at the optimal temperature (*p* < 0.01 and *p* < 0.001) and even lower at 4 °C (*p* < 0.001). Even though 37 °C is the optimal temperature for staphylococci growth, other studies obtained stronger *S. aureus* biofilm at 25 °C which is in accordance with our results [33,34]. Accordantly, Malheiros et al. also showed that the adherence of *S. aureus* on polyethylene was higher at 20 °C in comparison to lower incubation temperatures of 7, 10, 12, and 15 °C [35]. Despite being significantly lower, staphylococci were able to form biofilm at 4 °C which may be explained by an increase in staphylococcal cell surface hydrophobicity as previously shown [33].

**Figure 3:**
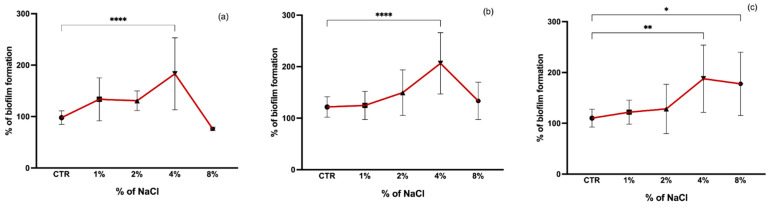
Biofilm formation capacity of MRSA (**a**), MSSA (**b**), and CoNS (**c**) under different salinity concentrations. Symbols represent the biomass mean of the biofilm formed at each NaCl concentration tested. Statistical significance was determined using Dunnett’s multiple comparison test against the control (CTR) conditions (* *p* < 0.05; ** *p* < 0.01; **** *p* < 0.0001).

It has been shown that the addition of NaCl may influence the biofilm formation of staphylococci [36,37]. In our study, a comparison of biofilm formation was made between control (biofilm formation without the presence of NaCl) and increasing concentrations of NaCl (1, 2, 4, and 8%) (Figure 3). The optimal concentration for biofilm formation was 4% NaCl for all isolates, with a significant difference from the control group (*p* < 0.001). CoNS seem to have a greater tolerance to NaCl since at a concentration of 8% there was no reduction in biofilm formation as marked as in *S. aureus*, which was also significantly greater than the biofilm formation without NaCl (*p* < 0.05). It has been shown that the presence of NaCl at concentrations of 4% and 6% increases staphylococci biofilm formation leading to an enhanced aggregation and biofilm stability conferred by the expression of the *ica* operon [38,39]. Mirani et al. showed that biofilm formation by a foodborne *S. aureus* increased at 7% of NaCl [40]. In contrast, in the study of Vaezi et al., concentrations above 6% of NaCl had an inhibitory effect on the biofilm formation of *S. aureus* [36]. It has been suggested that the induction of biofilm formation by NaCl is more pronounced in MSSA than MRSA strains [41]. However, in our study, a lower concentration of NaCl of 1% led to a more pronounced increase in biofilm formation in MRSA strains than in MSSA which is in agreement with what was obtained by Lade et al., who showed that low concentrations of NaCl (1.0% and 2.0%) induced biofilm formation more effectively for MRSA than MSSA strains [42]. The enhanced staphylococci biofilm formation at low concentrations of NaCl is due to the activation of the *ica*ADBC operon which results in polysaccharide intercellular adhesin (PIA) production necessary for biofilm formation and stability [41,42].

**Figure 4:**
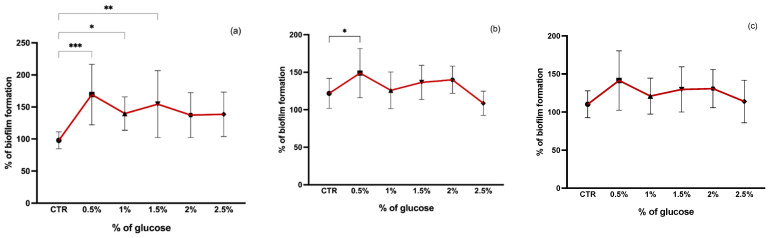
Biofilm formation capacity of MRSA (**a**), MSSA (**b**), and CoNS (**c**) under different glucose concentrations. Symbols represent the biomass mean of the biofilm formed at each glucose concentration tested. Statistical significance was determined using Dunnett’s multiple comparison test against the control (CTR) conditions (* *p* < 0.05; ** *p* < 0.01; *** *p* < 0.001).

Glucose concentration also seems to affect biofilm formation. In our study, we tested the effect of glucose at 0.5, 1, 1.5, 2, and 2.5% on staphylococci biofilm formation and found that 0.5% of glucose was the optimal concentration for biofilm formation in all isolates (Figure 4). TSB supplemented with 0.5% of glucose significantly promoted the biofilm formation of both MSSA and MRSA isolates when compared to TSB alone (*p* < 0.05 and *p* < 0.001, respectively). Glucose appears to have a greater influence on biofilm formation of MRSA strains than on MSSA strains since there was a significant increase in biofilm production at concentrations of 1% and 1.5% of glucose (*p* < 0.05 and *p* < 0.01, respectively) which suggests that the rate of biofilm formation induced by glucose is distinct for MRSA and MSSA strains as shown in other studies [42]. Although there was a slight increase in CoNS biofilm production after the addition of glucose, this was not significant for any of the concentrations. Similar results were reported in other studies where the addition of glucose to the medium had no effect on CoNS biofilm production [43]. Furthermore, it has been suggested that glucose has a negative effect on the *ica*ADBC gene expression in CoNS and the glucose added to the medium may lead to cells gaining more energy and so biofilm formation for protection is not necessary [43]. In addition, it has been shown that the presence of glucose alters the patterns of proteins in the extracellular matrix (ECM) promoting or suppressing the expression of some ECM proteins [44]. The presence of glucose also represses the *agr* quorum sensing leading to a decrease in pH due to short-chain fatty acids excretion resulting from glucose metabolism [44,45].

**Figure 5:**
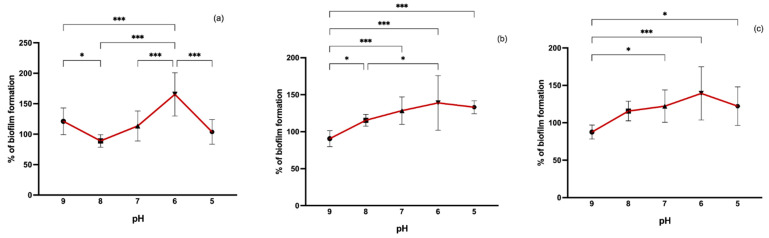
Biofilm formation capacity of MRSA (**a**), MSSA (**b**), and CoNS (**c**) under different pH values. Symbols represent the biomass mean of the biofilm formed at each pH value. Statistical significance was determined using Tukey’s multiple comparison test (* *p* < 0.05; *** *p* < 0.001).

In our study, we also evaluated the effect of pH variation on biofilm formation. Differences in pH values varied greatly between MRSA, MSSA, and CoNS (Figure 5). However, biofilm formation was more induced at a pH value of 6 for all isolates. In surface water isolates, both MSSA and CoNS, at a more basic pH (9), biofilm formation was significantly lower than at other pH values. In MRSA from wastewater, biofilm formation was enhanced at pH 9 compared to pH 8 and 5. It has been shown that lower pH values may lead to weaker biofilm formation [44,45]. However, neither a very acidic nor a very alkaline medium promotes biofilm formation [46]. Supporting our results, studies have revealed that there is a higher biofilm production at weak acidic pH values when compared to basic pH [47]. It has been shown that acidic pH values prompt functional amyloid assembly promoting biofilm formation [48].

The influence of aerobic and anaerobic conditions on biofilm formation. All isolates produced significantly less biofilm biomass under anaerobic than aerobic conditions (*p* < 0.05). Some studies have analyzed the staphylococci biofilm formation under anaerobic than aerobic conditions and the reported results vary widely. Stepanović et al. showed that biofilm formation under anaerobic conditions did not differ from biofilm formation under aerobic conditions [49]. Other studies have reported that biofilm formation under anaerobic conditions is enhanced probably due to the expression of PIA, teichoic acids, and proteins important for biofilm production [50,51]. In accordance with our results, Asai et al. showed that biofilm formation of staphylococci grown under anaerobic conditions was significantly lower than that produced under aerobic conditions, suggesting that PIA production was induced under aerobic conditions [52].

**Figure 6:**
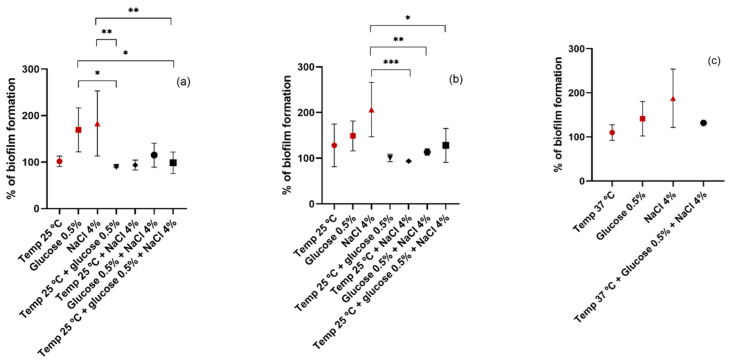
Biofilm formation capacity of MRSA (**a**), MSSA (**b**), and CoNS (**c**) under different conditions. Symbols represent the biomass mean of the biofilm formed at different conditions or combination of conditions. Statistical significance was determined using Tukey’s multiple comparison test (* *p* < 0.05; ** *p* < 0.01; *** *p* < 0.001).

Finally, the combination of more than one condition together was investigated. Regarding the choice of parameters to combine, the conditions were selected with which the highest biofilm biomass was obtained for temperature, glucose, and NaCl, namely, 25 °C, 0.5%, and 4%, respectively, for the MRSA and MSSA strains, and 37 °C, 0.5%, and 4%, respectively, for the CoNS strains. Since the ideal temperature for biofilm growth of CoNS isolates was already 37 °C, it would only make sense to combine the NaCl and glucose conditions. The results are shown in Figure 6. The results obtained in the previous experiments were included to facilitate comparison and are represented in dark red. For all isolates, the combination of different affects negatively the biofilm formation when compared to the results obtained by each isolated condition. Regarding MRSA isolates, there are significant differences in biofilm production when comparing the conditions of 0.5% glucose and 4% NaCl with the combination of these parameters. In MSSA strains, the combination of 4% NaCl with temperature and glucose leads to a significant decrease in biofilm production when compared to 4% NaCl alone. As for the CoNS isolates, the combination of 4% NaCl and 0.5% glucose led to the production of a smaller amount of biofilm biomass than NaCl and glucose separately. However, although the difference was not statistically significant, with the combination of these two conditions there was a greater production of biofilm than that obtained at a temperature of 37 °C without the addition of NaCl and glucose. Our results differ from the results obtained in the study of Rode et al. in which the combination of NaCl and glucose enhanced the biofilm formation of most *S. aureus* strains [53]. However, the concentrations of NaCl and glucose used in that study were different from ours. On the other hand, the results obtained by Vázquez-Sánchez et al. are in accordance with ours. In that study, no synergy was noted between the addition of glucose and NaCl. In addition, a negative correlation was observed with biofilm formation and the addition of both substances [6].

## 4. Conclusions

Our results indicate that multiple environmental factors, including temperature, pH, glucose, salinity, and oxygen, induce stress responses and can influence the biofilm formation of staphylococci isolates. Significant differences were detected among MRSA, MSSA, and CoNS isolates. However, for some conditions, all isolates followed the same pattern. Further studies will be carried out in order to understand the mechanisms underlying biofilm formation under different conditions, particularly in CoNS, which are much less studied than *S. aureus* strains.

## Data Availability

Not applicable.

## Supplementary Materials

The following supporting information can be downloaded at: https://www.mdpi.com/article/10.3390/pathogens11101069/s1, Table S1: Absorbance values obtained at 570 nm.
